# Comparing human–*Salmonella* with plant–*Salmonella* protein–protein interaction predictions

**DOI:** 10.3389/fmicb.2015.00045

**Published:** 2015-01-28

**Authors:** Sylvia Schleker, Meghana Kshirsagar, Judith Klein-Seetharaman

**Affiliations:** ^1^Klein-Seetharaman Laboratory, Division of Metabolic and Vascular Health, Warwick Medical School, University of Warwick, Coventry, UK; ^2^Department of Molecular Phytomedicine, Institute of Crop Science and Resource Conservation, University of Bonn, Bonn, Germany; ^3^Language Technologies Institute, School of Computer Science, Carnegie Mellon University, Pittsburgh, PA, USA

**Keywords:** host–pathogen interactions, systems biology, prediction, pathways, interactome

## Abstract

Salmonellosis is the most frequent foodborne disease worldwide and can be transmitted to humans by a variety of routes, especially via animal and plant products. *Salmonella* bacteria are believed to use not only animal and human but also plant hosts despite their evolutionary distance. This raises the question if *Salmonella* employs similar mechanisms in infection of these diverse hosts. Given that most of our understanding comes from its interaction with human hosts, we investigate here to what degree knowledge of *Salmonella*–human interactions can be transferred to the *Salmonella*–plant system. Reviewed are recent publications on analysis and prediction of *Salmonella*–host interactomes. Putative protein–protein interactions (PPIs) between *Salmonella* and its human and *Arabidopsis* hosts were retrieved utilizing purely interolog-based approaches in which predictions were inferred based on available sequence and domain information of known PPIs, and machine learning approaches that integrate a larger set of useful information from different sources. Transfer learning is an especially suitable machine learning technique to predict plant host targets from the knowledge of human host targets. A comparison of the prediction results with transcriptomic data shows a clear overlap between the host proteins predicted to be targeted by PPIs and their gene ontology enrichment in both host species and regulation of gene expression. In particular, the cellular processes *Salmonella* interferes with in plants and humans are catabolic processes. The details of how these processes are targeted, however, are quite different between the two organisms, as expected based on their evolutionary and habitat differences. Possible implications of this observation on evolution of host–pathogen communication are discussed.

## INTRODUCTION

*Salmonella* are Gram-negative bacteria comprising more than 2500 known serovars (Abraham et al., 2012). The pathogen has an unusually broad host range infecting diverse species, including humans, sheep, cows, reptiles, and plants. *Salmonella* causes severe worldwide health problems in developing as well as developed countries and constitutes one of the main causes of foodborne diseases in humans (World Health Organization, 2014). The bacteria can be transmitted to humans, e.g., through infected animals, contaminated meat, fish, and egg products, water, vegetables, and fruits. Diseases caused by *Salmonella* are divided into typhoid fever which is caused by *S.* Typhi and *S.* Paratyphi and Salmonellosis (diarrheal diseases) caused by a variety of non-typhoidal *Salmonella* (NTS) serovars, mainly *S*. Enteritidis and *S*. Typhimurium. In the USA alone, more than one million cases are reported annually (Centers for Disease Control and Prevention, 2014b). Besides reports on *Salmonella* outbreaks due to the consumption of contaminated animal products, there are many cases where an outbreak was ascribed to contaminated vegetables and fruits or processed plant products. For instance, in 2011, the consumption of mung bean sprouts lead to a Salmonellosis outbreak in Germany (Abu Sina et al., 2012). In the USA, 25% of all reported multistate outbreaks of *Salmonella* in 2012 and 2013 have been linked to plant products, e.g., peanut butter, mangoes, and cucumbers (Centers for Disease Control and Prevention, 2014a).

While *Salmonella* may not require active invasion of plant tissue as part of its transmission mechanism, it is now well established that they are able to invade plant cells and proliferate inside plants (Schikora et al., 2008). This makes plants a bona fide host for *Salmonella*, and poses the question to what extent the communication between *Salmonella* and its plant host are related to the better established interaction with the human host. One means of communication between any pathogen and its hosts are via protein–protein interactions (PPIs). *Salmonella*–human interactions are much better studied than *Salmonella*–plant interactions, and the question is to what extent the knowledge can be transferred from the human to the plant system. For its mammalian hosts, it is known that *Salmonella* exerts its pathogenicity by injection of proteins, called effectors, into the host cell. These effectors interact with host proteins and thereby influence host mechanisms for the pathogen’s benefit. The best characterized set of *Salmonella* effectors are those encoded on *Salmonella* pathogenicity islands 1 and 2 (SPI-1 and -2). These gene clusters contain the genetic information of the proteins building the type three secretion systems 1 and 2 (TTSS-1 and -2) which *Salmonella* utilizes to translocate the SPI-1 and -2 encoded effectors into the host cell. These effectors then interact with host proteins, the host–pathogen interactome, which is of major importance for the functional interplay between the two organisms.

Here, we will first review the evidence for experimentally demonstrated functional interactions between *Salmonella* proteins and plant cellular targets (see Evidence for Plants as Bona Fide Hosts for *Salmonella*). We will then briefly summarize the current state of knowledge on the *Salmonella*–human interactions (see Known *Salmonella*–Human Protein–Protein Interactions), which is the best studied host organism and has been extensively reviewed elsewhere (Haraga et al., 2008; McGhie et al., 2009; Heffron et al., 2011; Schleker et al., 2012b). Because the number of known interactions, even for human, is still small, we will then review recent results on the predicted *Salmonella*–human entire interactome (see The *Salmonella*–Human Predicted Interactome), followed by the extrapolation to the plant host (see The *Salmonella*–*Arabidopsis* Predicted Interactome). We will then compare the *Salmonella*–plant interactome with available plant experimental data (see Comparison of Plant–*Salmonella* PPI Prediction Results with Experimental Data) and finally compare the two host (plant and human) responses with each other (see Comparison of Plant and Human Host Responses to *Salmonella* Challenge).

## EVIDENCE FOR PLANTS AS BONA FIDE HOSTS FOR *Salmonella*

Although *Salmonella* is better known as a human and animal pathogen, and the majority of human infections are incurred via animal product routes such as meat and eggs, there is growing evidence that *Salmonella* actively interacts with plants and utilizes these organisms as alternative hosts making *Salmonella* a true plant pathogen. Infection studies coupled to fluorescence microscopy demonstrated that *Salmonella* actively invades the interior of alfalfa sprouts (Gandhi et al., 2001; Dong et al., 2003), lettuce (Klerks et al., 2007b) and enters *Arabidopsis* cells and propagates there (Schikora et al., 2008). While some infected plants may not always show symptoms of infection (Gandhi et al., 2001; Charkowski et al., 2002; Islam et al., 2004; Barak et al., 2005; Klerks et al., 2007a; Lapidot and Yaron, 2009; Noel et al., 2010; Shirron and Yaron, 2011), a reduction in biomass production, wilting, chlorosis, and death of infected organs has clearly shown that plants can exert disease symptoms due to *Salmonella* infection (Klerks et al., 2007b; Schikora et al., 2008). Furthermore, contact of *Salmonella* with plants activates plant defense responses and the expression of pathogenesis-related (PR) genes. Flagella of *S.* Typhimurium are recognized by plants and the bacteria activate salicylic acid (SA)-dependent and -independent defense responses (Iniguez et al., 2005). Moreover, kinase activity assays and qRT-PCR analysis revealed that *S.* Typhimurium activates mitogen-activated protein kinase (MAPK) cascades in *Arabidopsis*. Furthermore, SA, jasmonic acid (JA), and ethylene (ET) signaling pathways contribute to the defense response (Schikora et al., 2008). Several PR genes are upregulated in infected plants including PR1, 2, and 4 and the plant defensin PDF1.2 (Iniguez et al., 2005; Schikora et al., 2008) with the enhanced expression of PR1 being dependent on a functional TTSS-1 (Iniguez et al., 2005). TTSS-1 and -2 effectors are clearly important for *Salmonella* pathogenicity in plants as evidenced by the observation that mutants compromised in these secretion systems proliferate slower in *Arabidopsis* compared to the wild type (WT). Further, these mutants seem to be unable to inhibit the plant hypersensitive response (HR; Schikora et al., 2011; Shirron and Yaron, 2011). Thus, functional TTSS-1 and -2 are necessary to suppress plant defense responses implying that *Salmonella* utilizes the same proteins to communicate with its mammalian and plant hosts.

## KNOWN *Salmonella*–HUMAN PROTEIN–PROTEIN INTERACTIONS

Protein–protein interactions constitute an important part of the communication between a host and its pathogen and thus, are fundamental to understanding the biological processes occurring during infection. Interactions between primarily human and *Salmonella* and their biological significance have been reviewed previously (Haraga et al., 2008; McGhie et al., 2009; Heffron et al., 2011; Schleker et al., 2012b). A recent extensive literature and data base survey, screening more than 2200 journal articles and over 100 databases, revealed that there is relatively little published information available: only 58 direct and 3 indirect PPIs between 22 *Salmonella* TTSS-1 and -2 effectors and 49 mammalian proteins (including 40 human proteins) could be retrieved by our literature survey (Schleker et al., 2012b). In the meantime two more interactions have been published adding two *Salmonella* effectors and two human proteins to the list of *Salmonella*–human interactions (Spano et al., 2011; Odendall et al., 2012). With these interactions *Salmonella* interferes with a variety of host cellular processes for its benefit. For instance, these PPIs trigger the modification of the actin cytoskeleton, recruitment of vesicles, the formation of the *Salmonella* containing vacuole (SCV) and tubulation and interfere with cytokine secretion, inflammatory response, antigen presentation by major histocompatibility complexes (MHC) I and II and apoptosis.

## THE *Salmonella*–HUMAN PREDICTED INTERACTOME

Because our knowledge of known *Salmonella*–mammalian interactions is limited, several publications describe the prediction of PPIs between *Salmonella* and human proteins. These include three interolog approaches where putative *Salmonella*–human PPIs are obtained by sequence and/or domain comparison to proteins of known interactions (Krishnadev and Srinivasan, 2011; Arnold et al., 2012; Schleker et al., 2012a). Thus, interolog approaches make use of available information from sequence, domain, and PPI databases. Whereas Krishnadev and Srinivasan (2011) used iPfam and DIP databases as information input to their approach, Arnold et al. (2012) used DIMA 3.0 and SIMAP databases and Schleker et al. (2012a) obtained domain information from iPfam and 3DID databases, protein sequence information from uniprot and known PPIs from BIANA that integrates available data from 10 PPI databases. Due to the fact that the retrieved list of putative *Salmonella*–human PPIs is unranked, techniques to filter the predictions are needed in order to obtain a subset of interesting and relevant PPIs. In the three interolog studies, the predicted interactions were filtered by different methods according to properties of the *Salmonella* protein (Krishnadev and Srinivasan, 2011; Schleker et al., 2012a). For example, these properties may include whether the protein contains a predicted transmembrane helix or an extracellular signal peptide (Krishnadev and Srinivasan, 2011), or the predicted list of human interaction candidates was ranked according to the degree of these candidates in the human intraspecies network thereby underlying the assumption that *Salmonella* effectors tend to interact with host hub proteins (Arnold et al., 2012). Another filtering approach was to apply the GUILD method (Guney and Oliva, 2012; Schleker et al., 2012a). GUILD is a genome-wide network-based prioritization framework based on known human disease genes and disease-gene prioritization algorithms. Thereby, GUILD indicates the putative human target proteins that are involved in human pathology and assigns a score to each target.

Conceptually different from the interolog approaches, Kshirsagar et al. (2012) described a supervised machine learning approach that integrates information from diverse sources, including but not limited to interolog information. Here, the set of known interactions (Schleker et al., 2012b) is used as gold standard and diverse data such as tissue expression, transcriptomic, gene ontology (GO), sequence, and domain information are used as features. In a so-called classification approach, a model is learnt from this gold standard and feature input to differentiate the two classes, interact and not interact. Because not all features are equally well studied, such integration is challenging and many features display what is known as the missing value problem, where values are available only for a subset of the interactions. Kshirsagar et al. (2012) therefore made use of techniques for missing value imputation in order to overcome the problem that often certain attributes are unavailable in the used data sources. Kshirsagar et al. (2012) used data from other related bacterial species, in addition to information from *Salmonella*. To transfer the information from the other closely related species to *Salmonella*, protein sequence alignment was carried out to define a measure of similarity between the proteins from the two species. Nearest-neighbor-based methods were then employed to combine such cross-species data. This approach proved superior to prediction techniques using generic imputation methods.

The best scored PPIs of the machine learning approach (score = 1) were with the *Salmonella* effectors SlrP and SspH2. Functional enrichment analysis for GO biological processes revealed that the putative human targets are involved in cellular processes related to, e.g., catabolic processes, proteolysis, cell death, regulation of transcription, regulation of kinase cascades, cytoskeleton organization, and cell cycle. Within the small subset of filtered PPIs obtained by Arnold et al. (2012) in the interolog approach, putative human interactors of SlrP and SspH2 are involved in the same processes as predicted by the machine learning approach. Whereas these studies concentrated on *Salmonella* TTSS-1 and -2 effectors, Krishnadev and Srinivasan, 2011 and Schleker et al. (2012a) additionally looked for putative interactions with any *Salmonella* protein and *Salmonella* virulence factor, respectively. The top 100 GUILD scored putative human targets were found to function in cell death, immune response, cytokine production and secretion, protein secretion, transport and localization, peptidase activity, and kinase cascades (Schleker et al., 2012a).

## THE *Salmonella*–*Arabidopsis* PREDICTED INTERACTOME

To our knowledge, to date there has been no report on the experimental identification of any interaction between a *Salmonella* effector protein and a plant protein. We therefore have to rely on predictions for the time being. The above described interolog approach was also utilized to predict interactions between *Salmonella* and *Arabidopsis* (Schleker et al., 2012a). The resulting putative interactome highlighted the *Salmonella* effectors SptP, SspH1, SspH2, and SlrP as the proteins with the highest number of interactions (hub proteins). Further, a comparison of the putatively targeted human and *Arabidopsis* proteins indicated that similar processes appear affected by the infection in these two hosts. Considering that the function of more than half of the *Arabidopsis* protein-coding genes is unknown, this comparison could help to elucidate the biological functions of so far uncharacterized *Arabidopsis* proteins, for example, to identify pathogen recognition receptors (Schleker et al., 2012a).

The *Salmonella*–*Arabidopsis* interactome has also been predicted by several machine learning approaches (Kshirsagar et al., 2015). As no data on known *Salmonella*–plant PPIs is available, this technique builds a prediction model based on the known *Salmonella*–human PPIs (Schleker et al., 2012b) and knowledge of plant interactions with other pathogenic bacteria such as *E. coli* (Kshirsagar et al., 2015). In one approach a two-step procedure involving kernel mean matching (KMM) and a support-vector-machine (SVM) based classifier model was applied to infer high confidence *Salmonella*–*Arabidopsis* predictions. The predictions of the five best models were aggregated by taking into account (a) the majority voting for a given protein pair to interact or not and (b) the average probability score indicating the confidence that a predicted PPI occurs. Some of the *Arabidopsis* proteins of high-scoring PPIs are predicted to interact with many different effectors. The number of predicted interactions by the KMM model is very large (∼160,000 when using a cut-off of 0.7; see Kshirsagar et al., 2015 and the full list of predictions is available in the supplementary of this paper). Even when using a very stringent cut-off of 0.9 there are ∼66,000 and 0.98 there are still ∼6200. Additionally, Kshirsagar et al. (2015) increased the stringency of the prediction model by exchanging the setting of the parameter “ratio between protein interact to non-interact” from 1:100 to 1:500 thereby obtaining a smaller subset of the predicted PPIs assumed to potentially occur with higher confidence (high class skew model). Because these predictions are not experimentally verified, it is very challenging to choose individual predictions. The choice is inherently biased by interest and expertise of the investigator. We have chosen specific pairs of our interest for which there was also supporting evidence in the literature from the predictions with the aim of demonstrating the utility of the predictions in generating biological hypothesis. Kshirsagar et al. (2015) had performed GO functional term enrichment analysis that has identified general trends, and we here further perform MapMan analysis for the set of ∼6200 to assist in analysis. From the general trends identified, we then chose specific pairs that are discussed in greater detail. For example, abscisic acid (ABA) insensitive 4 (ABI4) (also predicted by the high class skew model), an ET-responsive transcription factor involved in ABA signaling leading to callose deposition and stomata closure. ABI4 has been reported to thereby mediate resistance against the necrotrophic pathogens *Alternaria brassicicola*, *Plectosphaerella cucumerina*, and *Sclerotinia sclerotiorum* (Guimaraes and Stotz, 2004; Ton and Mauch-Mani, 2004). In the next section, we further interpret the prediction results from the KMM–SVM model from a biological point of view by comparing with published experimental data.

## COMPARISON OF PLANT–*Salmonella* PPI PREDICTION RESULTS WITH EXPERIMENTAL DATA

Here, we provide a discussion on the biological significance of the available KMM–SVM model predictions (Kshirsagar et al., 2015) based on comparison with available experimental data. In particular, Schikora et al. (2011) had analyzed the transcriptomic response of *Arabidopsis* plants infected with four different bacteria: *S.* Typhimurium WT, a *prgH*^–^ mutant with deficiency in expression of TTSS-1 genes, *Pseudomonas syringae*, and *E. coli* DH5alpha. About 30 *Arabidopsis* genes were exclusively differentially regulated upon infection with *Salmonella*. This included, for example, cytoskeleton-associated proteins. When comparing *Salmonella* WT and the *prgH*^–^ mutant, a large portion of *Arabidopsis* genes specifically upregulated in the *prgH*^–^ mutant were involved in the ubiquitin-dependent protein degradation process as well as cell wall, defense response and WRKY transcription factor clusters. The KMM–SVM classification approach predicts that proteins involved in these processes are putative targets of *Salmonella* effectors (Kshirsagar et al., 2015). Examples of how *Salmonella* interferes with plant defense response, gene activation, and plant metabolism are described below.

### *Salmonella* INTERFERES WITH PLANT BASAL DEFENSE RESPONSE AND GENE ACTIVATION

A basic plant defense response mechanism is the induction of MAPK cascades as well as Ca^2^^+^ influx into the cell upon recognition of flg22 by the FLS2 (flagellin-sensing 2) receptor. Kinase activation results in phosphorylation of WRKY transcription factors and thus, in the induction of defense genes. Ca^2^^+^ leads to the activation of Ca^2^^+^-dependent protein kinases (CDPKs) 4, 5, 6, 11 and the NADPH-oxidase RbohD (respiratory burst oxidase homolog protein D) that triggers a reactive oxygen species (ROS) burst (Segonzac and Zipfel, 2011; Gao and He, 2013). MAPK-dependent activation of the plant defense response against *S.* Typhimurium was demonstrated to be important because MPK3, MPK4, and MPK6 are rapidly activated upon *Salmonella* infection, and *mpk3* and *mpk6* mutants reveal accelerated susceptibility toward *S.* Typhimurium (Schikora et al., 2008). Further, genes coding for calcium-binding proteins have higher expression levels upon *Arabidopsis* infection with a *Salmonella prgH* deficiency mutant compared to the WT (Schikora et al., 2011). Several CDPKs, calcium-binding proteins and (putative) WRKYs are predicted to be targeted by *Salmonella* with the KMM–SVM modeling approach, for instance, CDPK4 and WRKY28. It has been shown that WRKY28 contributes to the induction of oxidative burst as well as the activation of SA-, JA-, and ET-dependent defense signaling pathways (Chen et al., 2013). All three hormone dependent pathways were shown to play a role in defense against *Salmonella* (Iniguez et al., 2005; Schikora et al., 2008).

Mitogen-activated protein kinases are known targets of bacterial effectors. For instance, MPK4 is phosphorylated by the *P. syringae* effector AvrB leading to the induction of the JA signaling pathway. This mechanism has been demonstrated to positively influence the growth of *P. syringae* (Cui et al., 2010). *P. syringae* effector HopF2 which exerts mono-ADP ribosyltransferase activity inhibits MKK5 activity (Wang et al., 2010). The KMM–SVM model predicts an interaction between *Salmonella* effectors and *Arabidopsis* mitogen-activated protein kinase kinase kinase ANP1 (Kshirsagar et al., 2015). ANP1 can be activated by H_2_O_2_ and induces the MPK3 and MPK6 signaling cascades thereby leading to the expression of defense-related genes (Kovtun et al., 2000).

Other transcription factors predicted to be targeted by *Salmonella* effectors are WRKY46, 53, and 70. Thilmony et al. (2006) found that the pathogen-associated molecular patterns (PAMP)-inducible transcription factor WRKY53 is significantly downregulated in *P. syringae* infected *Arabidopsis* plants thereby interfering with WRKY53-dependent cellular mechanisms. WRKY53 plays an important role in regulation of plant senescence and defense response as the transcription factor can be directly phosphorylated by MEKK1 (Zentgraf et al., 2010). *Arabidopsis* mutants in *wrky*46, 53, and 70 revealed increased susceptibility toward *P. syringae* leading the authors to the conclusion that these three transcription factors have an overlapping or synergistic role in regulating defense response against *P. syringae* (Hu et al., 2012).

### *Salmonella* IMPACT ON PLANT METABOLISM

**FIGURE 1:**
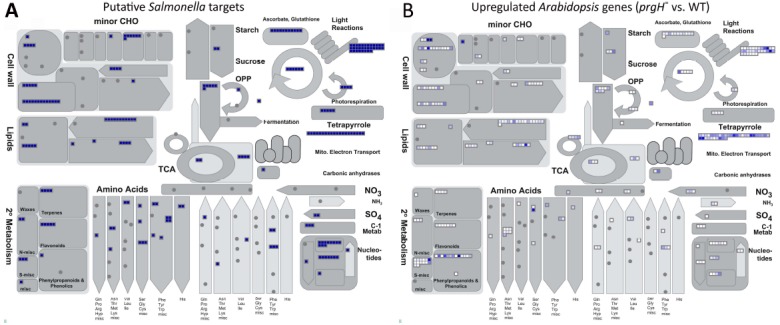
**Overview of metabolic processes putatively targeted and known to be repressed by *S.* Typhimurium effectors.** MapMan (Thimm et al., 2004) analysis providing a metabolism overview of **(A)** predicted *Arabidopsis* targets of *S.* Typhimurium effectors predicted with cut-offs of 1 (voting score) and 0.98 (probability aggregated score) by the KMM–SVM model (Kshirsagar et al., 2015) and **(B)** *Arabidopsis* genes experimentally identified to be upregulated upon infection with *S.* Typhimurium *prgH*^–^ vs. WT (Schikora et al., 2011). Each small square displays a predicted *Arabidopsis* target of *S.* Typhimurium **(A)** or an upregulated *Arabidopsis* gene **(B)**. In **(B)**, the color intensity visualizes the degree of upregulation.

The KMM–SVM predictions indicate that *S.* Typhimurium effectors heavily target *Arabidopsis* metabolic and biosynthetic processes (Kshirsagar et al., 2015). For visualization, we utilized MapMan (Thimm et al., 2004), a software tool that assigns *Arabidopsis* proteins to specific plant processes and pathways, to see what metabolic pathways are putatively interfered with by *S.* Typhimurium effectors. A comparison with available transcriptomic data revealed a clear overlap in the pathways predicted to be targeted by *S.* Typhimurium effectors (Figure 1A) and those identified to involve genes that are upregulated upon *S.* Typhimurium *prgH*^–^ vs. WT infection (Figure 1B; Schikora et al., 2011). In Figure 1A, every small blue square displays one *Arabidopsis* protein predicted by KMM–SVM to be targeted by one or more *S.* Typhimurium effectors. In Figure 1B, *Arabidopsis* genes known to be upregulated during *S.* Typhimurium *prgH*^–^ vs. WT infection are visualized by white to blue small squares depending on the degree of upregulation. Thus, the darker blue the square is, the more efficiently this gene is suppressed by *S.* Typhimurium TTSS-1 effectors. In conclusion, the metabolic processes *S.* Typhimurium most intensively interferes with include those related to the cell wall, lipids, light reactions, tetrapyrrole, and secondary metabolism of, e.g., terpenes (Figure 1B).

The impact of the pathogen on plant metabolism may be general as parallels can also be found in available data for another pathogen, *P. syringae*. Thilmony et al. (2006) analyzed the transcriptional response of *Arabidopsis* infected with *P. syringae* WT and mutants with deficiencies in expressing COR (coronatine) toxin and/or *hrp*-dependent TTSS effectors. Many of the identified TTSS effector regulated *Arabidopsis* genes are involved in metabolic processes similar to those described for *Salmonella* above. Especially cell wall genes, genes involved in photosynthesis and the Calvin cycle are repressed but also genes involved in secondary metabolism related to phenylpropanoids and terpenes.

## COMPARISON OF PLANT AND HUMAN HOST RESPONSES TO *Salmonella* CHALLENGE

One major difference between humans and plants is that plant cells have a cell wall and mammalian cells do not. Thus, the question arises, how *Salmonella* can overcome this barrier and enter the cytoplasm. Schikora et al. (2008) describe the presence of green fluorescent protein (GFP)-labeled *S.* Typhimurium inside *Arabidopsis* root cells and intact protoplasts 3 and 20 h after infection, thereby demonstrating that *Salmonella* can enter the plant cytoplasm and proliferate there. Plant pathogenic bacteria are known to target the plant cell wall by a variety of enzymes with hydrolytic activity. For example, *Pseudomonas viridiflava* secretes a pectate lyase (Jakob et al., 2007) and *Xanthomonas oryzae* produces a xylanase to degrade the cell wall (Rajeshwari et al., 2005). Both are necrotrophic bacterial pathogens. On the other hand, there are reports showing that bacteria can be found inside intact plant cells. For instance, Quadt-Hallmann et al. (1997) detected the endophytic bacterium *Enterobacter asburiae* inside cotton root cells 6 h after inoculation and hydrolysis of cellulose at these sites. Another report demonstrated the colonization of banana periplasm and cytoplasm of intact cells by endophytic bacteria (Thomas and Sekhar, 2014). Endosymbiotic bacteria, e.g., Rhizobacteria, are taken up into the plant cytoplasm and are engulfed inside a membrane of plant origin, called the symbiosome membrane. There is evidence that the engulfed organism actively inhibits its degradation through plant cellular mechanisms (Parniske, 2000). This procedure of bacterial internalization and active suppression of degradation reveals similarities to human bacterial pathogens like *Salmonella*. For the human host, the best described process of how *Salmonella* enters the cell is driven by the TTSS-1 effectors SipA, SipC, SopA, SopB, SopD, SopE2, and SptP. These effectors induce modification of the actin cytoskeleton, promote membrane ruffling, recruitment of vesicles to the site of *Salmonella* internalization and rearrangement of the cytoskeleton to its normal shape after engulfment of the pathogen in the SCV (Schleker et al., 2012b). To our knowledge, it is unknown, how *Salmonella* overcomes the cell wall barrier. The pathogen may secrete cell wall hydrolyzing enzymes and/or interfere with cell wall biogenesis, or it enters a necrotrophic phase and thus kills the plant cells. On the other hand it could as well be that the invaded plant cell stays intact or preferential infection occurs when plant material decays through other processes (Schikora et al., 2011). In favor of the presence of cell wall-modifying mechanisms is evidence from the KMM–SVM modeling approach. It predicts the interaction of *Salmonella* effectors with *Arabidopsis* proteins involved in cell wall organization, e.g., xyloglucan endotransglucosylase proteins that are involved in cell wall construction of growing cells. Moreover, *Arabidopsis* proteins of the Arp2/3 complex that mediates actin polymerization and Actin-11, for instance, are putative *Salmonella* effector targets involved in cytoskeleton organization (Kshirsagar et al., 2015).

### *Salmonella* EFFECTORS INTERFERE WITH HOST UBIQUITIN PROTEASOME MECHANISMS IN BOTH ORGANISMS

As can be seen in the high-scored KMM–SVM predictions, experimental data and the known human targets of *Salmonella* effectors, host ubiquitin-related cellular processes seem to be targeted by *Salmonella* in both, human and plant hosts.

One central eukaryotic regulatory cellular mechanism involves the attachment of ubiquitin or ubiquitin-like proteins (Ub) to target molecules generally through an enzyme cascade comprising Ub activation by E1, Ub conjugation by E2 and Ub-substrate ligation by E3. In *Arabidopsis* over 5% of the proteome are proteins involved in the ubiquitination machinery demonstrating its importance (Smalle and Vierstra, 2004). Hundreds of human and plant E3 ligases are proposed to exist which belong to several different known types of E3 ligases which include RING-finger, HECT, and U-box type single protein E3 ligases as well as multi subunit RING-finger type E3 ligases comprising Cullin-RING and APC/C (anaphase promoting complex/cyclosome) E3 ligases (Zeng et al., 2006; Vierstra, 2009). E3 ligases comprise an E2 binding domain (HECT, U-box, RING-finger) and a target recognition subunit, e.g., F-box. In case of multi subunit E3 ligases additionally a Cullin, an adapter protein and for APC/C other subunits are present. Substrates can be mono- or polyubiquitinated with diverse linkage types between the Ub proteins. The modified proteins are either targeted for proteasomal degradation or play a functional role in diverse processes, e.g., endocytosis, gene expression, DNA repair or NF-κB activation in mammalian cells and, e.g., hormone signaling and defense response in plants.

It is known that pathogens exploit the mammalian and plant Ub system to enable their invasion and survival but so far only a very limited number of host–pathogen interactions within this highly important regulatory process have been described (Groll et al., 2008; Duplan and Rivas, 2014). All of these known interactions of *Salmonella* are with the mammalian Ub machinery whereas nothing is known on the plant side. There is a large space of putative interactions that remains to be elucidated based on the fact that already four *Salmonella* effectors are experimentally confirmed E3 ligases (SopA, SlrP, and SspH1 and 2). SopA, a HECT-like E3 ligase, which has been shown to interact with the E2 UbcH7, is known to induce polymorphonuclear neutrophil migration, a process attributed to its Ub ligase activity. However, so far the host targets are unknown (Zhang et al., 2006). The effector itself is ubiquitinated by the host E3 ligase RMA1. Monoubiquitinated SopA is assumed to be involved in *Salmonella* escape from the SCV and polyubiquitinated SopA is degraded by the host proteasome (Zhang et al., 2005). The E3 ligase SlrP has been shown to ubiquitinate thioredoxin 1 (Bernal-Bayard and Ramos-Morales, 2009) and to interact with ERdj3 (endoplasmic reticulum DNA J domain-containing protein 3; Bernal-Bayard et al., 2010). Both interactions are supposed to induce cell death. It is speculated that the interaction of SlrP with ERdj3 contributes to the inhibition of antigen presentation by MHC-I (Granados et al., 2009; Bernal-Bayard et al., 2010). The interaction of SspH1, an effector possessing E3 ligase activity, with PKN1 (serine/threonine-protein kinase N1) is proposed to inhibit NF-κB and thus IL-8 (interleukin-8) secretion (Haraga and Miller, 2006). SspH2 has been shown to interact with the E2 UbcH5 and to synthesize K48-linked Ub chains and thus likely targets its substrates for proteasomal degradation (Levin et al., 2010). SspH2 binds filamin A and profilin-1 thereby preventing cross-linking of F-actin and polymerization (Miao et al., 2003). Moreover, SspH2 has been reported to interact with Sgt1 (suppressor of G2 allele of SKP1 homolog), AIP (AH receptor-interacting protein), Bub3 (mitotic checkpoint protein BUB3), 14-3-3γ (protein kinase C inhibitor protein 1), and BAG2 (BAG family molecular chaperone regulator 2) but functions of these interactions are not known (Auweter et al., 2011). In addition to this, AvrA and SseL are deubiquitinating *Salmonella* enzymes that cleave Ub from IκBα and thereby inhibit NF-κB-mediated gene expression (Ye et al., 2007; Le Negrate et al., 2008). Recently, GogB, an effector mimicking a eukaryotic F-box, has been shown to inhibit NF-κB activation through its interaction with a host SCF (Skp-Cullin-F-box) E3 ligase (Pilar et al., 2012). As, for instance, it has been demonstrated that *Salmonella* enhances the internalization of MHC-II antigens by a ubiquitination event through a so far not identified effector it is possible that other *Salmonella* E3 ligases remain to be discovered (Lapaque et al., 2009). Furthermore, it can be presumed that many substrates of the known *Salmonella* E3 ligases are unknown. Moreover, host Ub mechanisms targeting *Salmonella* effectors as well as *Salmonella* effectors that mimic other proteins of the host Ub system, e.g., F- or U-box proteins, remain to be found.

Although to date there have not yet been any experimentally confirmed interactions of *Salmonella* effectors with the plant Ub system, several reports indicate an important role of the plant Ub proteasome system in pathogen defense response in general. About 50 genes involved in the Ub-dependent degradation pathway have been found to be upregulated upon inoculation of *Arabidopsis* with a *S.* Typhimurium *prgH*^–^ mutant compared to the WT control. Thus, *Salmonella* TTSS-1 effectors hinder the expression of these genes which mainly code for E2 and RING ligases (Schikora et al., 2011). It has been demonstrated that TTSS effectors of other bacterial pathogens also interact with the plant Ub proteasome machinery. One example is the group of *Ralstonia solanacearum* GALA effectors which are LRR (leucine-rich repeat) F-box proteins that interact with SKP1-like proteins (Angot et al., 2006). Secondly, *P. syringae* HopM1 targets *Arabidopsis* proteins and induces their proteasomal degradation. One of these proteins is the guanine nucleotide exchange factor MIN7, which plays a role in vesicle trafficking (Nomura et al., 2006).

The KMM–SVM modeling approach by Kshirsagar et al. (2015) predicts *Salmonella* effectors to interact with E3 ligases, RING proteins, F- and U-box domain containing proteins. The *Salmonella* E3 ligase SspH2 and the deubiquitinase SseL are predicted to interact with a variety of *Arabidopsis* proteins including those, e.g., involved in transcriptional regulation, stimulus and defense response, biosynthetic and metabolic processes, RNA processing, protein localization and transport.

## CONCLUSIONS AND FUTURE WORK

Due to the lack of direct experimental data on *Salmonella*–*Arabidopsis* and limited data on the *Salmonella*–human interactions, we here utilized published predictions of these interactomes. This is of course a limitation as we cannot ascertain the reliability of the predictions without further testing. However, the predictions for human were statistically evaluated quantitatively using the gold standard data, giving us some confidence. One popular measure for evaluation is the F1 score, which can be interpreted as a weighted average of the precision and recall, where an F1 score reaches its best value at 1 and worst score at 0. For our *Salmonella*–human predictions the F1 score was 74 (Kshirsagar et al., 2012). This value compares favorably with other performances, such as for HIV-human interactions reported at 54 (Dyer et al., 2011). We cannot do this for *Arabidopsis*, but the overlap between our predictions and previous experimental studies especially using transcriptomics and cell signaling support the biological relevance of the predictions. However, often the same interactions are predicted for several *Salmonella* effectors, hinting at the functional importance of the host protein targeted, but the actual pairs of interacting proteins may not be true. Furthermore, missing information for many proteins makes it challenging to generate predictions for such proteins. This introduces the bias of predicting interactions for relatively better studied proteins and pathways. Ultimately, only the experiments can verify if a predicted interaction is real or not. The benefit in the prediction approach lies in the generation of biological hypotheses that are experimentally testable, vastly narrowing the search space for new interactions.

From the current state of knowledge it is also difficult to judge how similar or different *Salmonella* is compared to a bona fide plant pathogen. Nevertheless, *Salmonella* triggers plant processes typical for plant pathogens as reviewed above. While interpreting the *Salmonella*–*Arabidopsis* predictions, we in some cases made comparisons with the plant pathogen *P. syringae* and its interaction with *Arabidopsis*. This is mainly due to the fact that the interaction between *P. syringae* and *Arabidopsis* is a well-studied model system for which data is available. Looking at the lifestyle of both bacteria, *Salmonella* as well as *P. syringae* colonize the plant apoplast and make use of TTSS and effectors. As we focus here on the interaction of *Salmonella* effectors with plant proteins, we think that comparing the interplay of both bacteria with *Arabidopsis* on this level is possible. One obvious difference between the two organisms in plants is that *Salmonella* has been found inside root cells and *P. syringae* only in the intercellular space. This may point at a difference in infection strategies. This is supported by a transcriptomic analysis where a small set of *Arabidopsis* genes is only differentially regulated in response to *Salmonella* but not *P. syringae* (Schikora et al., 2011). Interestingly, *Pseudomonas* bacteria (most likely non-pathogenic) have been detected in the plant cytosol (Pirttilä et al., 2000) indicating that bacteria of this genus are capable of entering the plant cell. Casadevall (2008) proposes that it might well be that intracellular survival of microbes is an ancient and common mechanism of microbes rather than an exception. Thus, it may be speculated that *P. syringae* lost the ability to invade the plant cytosol and/or the plant evolved effective means to prevent internalization.

The predictions, known interactions and experimental data reviewed here indicate that *Salmonella* partly targets the same cellular processes in both hosts. For example, *Salmonella* effectors interfere with proteins of the ubiquitin degradation pathway in *Arabidopsis* and human and moreover, *Salmonella* effectors are E3 ligases, deubiquitinases, and F-box mimicking enzymes with specific functions in both hosts. Although the same type of cellular machinery is targeted and utilized, *Salmonella* seems to do it differently in human compared to *Arabidopsis*. For example, as reviewed above, in the human host *Salmonella* inhibits the immune response, e.g., by interfering with transcription factor NF-κB mediated gene expression, whereas in *Arabidopsis* expression of defense-related genes may be suppressed by targeting plant transcription factors like ERF2 and ERF094 (ET responsive transcription factor 2 and ERF094) as obtained from the predictions (also predicted by high class skew model). Differences in the way of interfering with the same cellular processes or achieving similar functions in both hosts may be due to several possibilities: (i) the bacterium has adapted to target those pathways differently in each host type, (ii) the bacterium acts elsewhere in the host, and the catabolic response to those changes is common to both hosts, (iii) the catabolic response is generic, and independent of bacterial action, (iv) the bacterium targets those pathways in one host, but not the other, but the second host’s response looks similar to the result of targeting that pathway in the first host (either as a result of targeting elsewhere, or a generic response).

When comparing interaction mechanisms of *Salmonella* with its human, animal, and plant hosts, one obvious question is, whether one of these was the primary host or whether a co-evolution occurred. It has been proposed that *Salmonella* evolution took place in five phases starting from a common ancestor about 25–40 million years ago (Bäumler et al., 1998; Porwollik et al., 2002). First, *Salmonella* separated from *E. coli*. This was accompanied by the acquisition of possibly about 500 genes including SPI-1 genes. Next, *S. enterica* diverged from *S. bongori* and gained—among others—the SPI-2 genes. In a third phase, diphasic *S. enterica* strains occurred and in phase four, *S. enterica* spp. I, which includes *S.* Typhi and *S.* Paratyphi, separated from the other spp. recruiting genes for adaptation to warm-blooded hosts. In a last phase, *S.* Typhimurium evolved and recruited about 140 genes the functions of which are mostly unknown. In total, more than 900 *S.* Typhimurium LT2 genes were identified that are not present in the genomes of the enterobacteria *E. coli* K12 and O157:H7, *K. pneumoniae* MGH 78578 and *Y. pestis* CO92 (Porwollik et al., 2002). Despite this evolutionary development, beside *S. enterica* other human-pathogenic enterobacteria like *E. coli* can colonize plants and thus use plants as vectors for transmission to its animal and human hosts (Jayaraman et al., 2014). It has been shown that during interaction of *S. enterica* and *E. coli* O157:H7 with *Medicago truncatula* about 30% of the plant genes are commonly regulated by both pathogens (Jayaraman et al., 2014) indicating that both pathogens to some extent provoke the same plant response. Barak et al. (2009) identified two *S. enterica* genes that play a role in swarming and biofilm formation and are important for plant colonization. Homologous genes have been found in plant-associated bacteria, e.g., *Agrobacterium tumefaciens*, *Ralstonia solanacearum*, and *P. syringae*, but not in *E. coli* (Barak et al., 2009). From a practical point of view it may be favorable for enteropathogenic bacteria like *Salmonella* to be able to use plants as secondary host for survival and transmission to its animal host. Thus, a co-evolution with both hosts would be likely. Interestingly, it has been reported that there is only a 5% overlap between the *Salmonella* promoters induced during infection of tomato compared to infection of macrophages (Teplitski et al., 2009) demonstrating that the majority of genes *Salmonella* utilizes in its animal and plant hosts are different. Moreover, there is evidence that the response of plants varies dependent on the *Salmonella* serovar that infects the plant (Berger et al., 2011) possibly indicating that genetic differences are the reason. Last but not least, it has been demonstrated that *Salmonella* is recognized by its plant hosts and the acquired TTSS-1 and -2 play an important role in suppressing plant defense response. Thus, it is obvious that *Salmonella* utilizes the same TTSS to interfere with defense response in its animal and plant hosts but so far it is uncertain which effectors *Salmonella* utilizes in the plant host. Further investigations will help to gain more insight into the evolution of *Salmonella* with respect to the pathogen’s ability to infect plants.

Beside the above-discussed difference that plants have and animal cells do not have a cell wall, another difference *Salmonella* has to face is the temperature. While the human body temperature is nearly constant around 37^°^C, the temperature of plants varies greatly according to the temperature of the environment. It is known that *Salmonella* is able to adapt to different environmental conditions by, e.g., sensing changes in pH and temperature and adjusting gene regulation accordingly. A variety of these regulatory mechanisms involve the expression of virulence genes. For instance, the promoters of *Salmonella* virulence genes *orgA, pagC, prgH,* and *spvA* reveal related sequence motifs to the *tlpA* promoter which is specifically activated upon temperature increase (Clements et al., 2001).

In conclusion, plants play an important role in the transmission of *Salmonella* infections and mounting evidence supports the notion that they constitute a bona fide host for *Salmonella*. As such, *Salmonella* can infect plants and initiates a two-way communication of plant response to invasion and *Salmonella* defense against this response and exploitation of resources. Communication is clearly via PPIs, but to date, the only known (experimentally confirmed) interactions involve mammalian proteins, especially human, and even those are very small in number due to the limited number of studies carried out in this field to date. We therefore focused this review on current approaches to prediction of the full interactome between human and *Salmonella* proteins and its extension to prediction of the interactome with *Arabidopsis* as a plant host. We find that there is significant overlap in the pathways predicted to be targeted by *Salmonella* in both hosts despite their evolutionary distance, while there are also distinct and host-specific responses. These involve in particular plant biosynthetic pathways not available in the human host and the complex human immune system response not available in plants. It is likely that the fundamental mechanisms of interference are highly related and particularly striking is the prevalence of predicted binding partners relating to the ubiquitin degradation system in both hosts. These predictions provide a rich source of experimentally testable hypotheses that can speed up scientific discovery of both the main-stream human host response as well as the niche host–pathogen interaction pair involving the newly discovered plant host for *Salmonella*.

### Conflict of Interest Statement

The authors declare that the research was conducted in the absence of any commercial or financial relationships that could be construed as a potential conflict of interest.
